# Establishing the intracellular niche of obligate intracellular vacuolar pathogens

**DOI:** 10.3389/fcimb.2023.1206037

**Published:** 2023-08-14

**Authors:** Tatiana M. Clemente, Rajendra K. Angara, Stacey D. Gilk

**Affiliations:** Department of Pathology and Microbiology, University of Nebraska Medical Center, Omaha, NE, United States

**Keywords:** *Anaplasma*, *Ehrlichia*, *Chlamydia*, *Coxiella*, pathogen vacuole, vesicular trafficking, egress, membrane

## Abstract

Obligate intracellular pathogens occupy one of two niches – free in the host cell cytoplasm or confined in a membrane-bound vacuole. Pathogens occupying membrane-bound vacuoles are sequestered from the innate immune system and have an extra layer of protection from antimicrobial drugs. However, this lifestyle presents several challenges. First, the bacteria must obtain membrane or membrane components to support vacuole expansion and provide space for the increasing bacteria numbers during the log phase of replication. Second, the vacuole microenvironment must be suitable for the unique metabolic needs of the pathogen. Third, as most obligate intracellular bacterial pathogens have undergone genomic reduction and are not capable of full metabolic independence, the bacteria must have mechanisms to obtain essential nutrients and resources from the host cell. Finally, because they are separated from the host cell by the vacuole membrane, the bacteria must possess mechanisms to manipulate the host cell, typically through a specialized secretion system which crosses the vacuole membrane. While there are common themes, each bacterial pathogen utilizes unique approach to establishing and maintaining their intracellular niches. In this review, we focus on the vacuole-bound intracellular niches of *Anaplasma phagocytophilum, Ehrlichia chaffeensis, Chlamydia trachomatis*, and *Coxiella burnetii*.

## Intracellular lifestyle

Unlike extracellular or facultative bacteria that thrive in diverse environments, obligate intracellular bacteria can only survive within the cytoplasm or vacuoles of host cells. This unique lifestyle shapes their interactions with the host and influences disease progression. By sequestering themselves inside the cytoplasm or membrane-bound vacuoles, these pathogens can evade detection and destruction by the host’s innate immune responses. This not only enables their survival but also provides them with a protected niche to replicate and spread within the host. However, obligate intracellular pathogens lack the ability to synthesize all the nutrients required for their survival. Many of these pathogens have undergone extensive genome reduction, leading to the loss of metabolic pathways and rendering them dependent on host cell resources for their essential needs. As a result, pathogen manipulation of the host cell is central to intracellular survival. In the case of vacuole-bound obligate intracellular bacterial pathogens, specialized secretion systems translocate bacterial effector proteins across the pathogen-containing vacuole membrane and into the host cytoplasm. *C. trachomatis* requires a Type III Secretion System (T3SS) for all stages of host cell infection, while *C. burnetii*, *A. phagocytophilum*, and *E. chaffeensis* utilize a Type IV Secretion System (T4SS). In addition, *C. burnetii* utilizes a Sec-mediated secretion system, while *A. phagocytophilum* and *E. chaffeensis* also secrete effector proteins through a Type I Secretion System (T1SS). These secretion systems are essential for establishing and maintaining the pathogen-containing vacuole through the action of effector proteins. These effector proteins utilize both enzymatic and non-enzymatic mechanisms to manipulate a wide range of host cell processes including vesicular trafficking, gene expression, and the innate immune response. Bacterial effector proteins often mimic eukaryotic protein structure and contain conserved domains and motifs such as ankyrin (Ank) and tetratricopeptide (TPR) repeats.

By unraveling the intricate mechanisms employed by these bacteria to colonize and survive within host cells, researchers can gain insights into host-pathogen interactions and identify potential targets for therapeutic interventions. Additionally, studying the unique adaptations of obligate intracellular bacteria can shed light on fundamental cellular processes and host immune responses. In this review, we will explore the fascinating world of vacuolar obligate intracellular bacteria, focusing on their lifestyle, challenges, and strategies employed to establish and maintain their intracellular niches. By examining notable examples such as *Anaplasma phagocytophilum, Ehrlichia chaffeensis, Chlamydia trachomatis, and Coxiella burnetii*, we aim to gain a comprehensive understanding of the complex dynamics between these pathogens and their host cells.

## Overview of pathogens and intracellular developmental cycles

### *Anaplasma phagocytophilum* and *Ehrlichia chaffeensis*

*Anaplasma phagocytophilum* and *Ehrlichia chaffeensis* are tick-borne rickettsia causing anaplasmosis and ehrlichiosis, respectively. *A. phagocytophilum* infects human neutrophils and causes human granulocytic anaplasmosis, while *E. chaffeensis* exclusively infects human monocytes and macrophages and causes human monocytic ehrlichiosis. The biphasic lifestyle of *A. phagocytophilum* and *E. chaffeensis* consist of the dense-core cell (DC, the infectious form) and the reticulate cell (RC, the replicative form). *A. phagocytophilum* and *E. chaffeensis* utilize caveolae and GPI-anchored proteins to gain entry into the host cell (Lin and Rikihisa, 2003b). Specifically, the *A. phagocytophilum* outer membrane protein (OmpA) binds the P-selectin glycoprotein ligand 1 (PSGL-1) receptor, while the bacterial proteins P44/Msp2, AipA, Asp14, Asp55, and Asp62 proteins are critical for internalization *via* unidentified mechanism (Herron et al., 2000; Park et al., 2003; Wang et al., 2006; Ge and Rikihisa, 2007; Seidman et al., 2014; Green et al., 2020). *E. chaffeensis* utilizes multiple adhesins for infection. EtpE (entry-triggering protein of *Ehrlichia*) binds the host GPI-anchored protein DNAse X, promoting bacterial entry through N-WASP-mediated actin polymerization, while TRP120 (tandem repeat protein 120) activates Wnt signaling to trigger bacterial entry (Mohan Kumar et al., 2015; Luo et al., 2016). Following internalization, both *A. phagocytophilum* and *E. chaffeensis* DC forms block lysosomal fusion with the phagosome. However, the mature pathogen-containing vacuoles have very different characteristics, with the *A. phagocytophilum* vacuole (ApV) containing markers for autophagosome, recycling endosomes, and multivesicular bodies and the *E. chaffeensis* vacuole (EcV) being more similar to an early endosome. *A. phagocytophilum* DC to RC differentiation occurs between 4 and 8 hours post infection, with the log phase of RC replication occurring over the next 20 hours. Between 28 and 36 hours post infection, the RC forms transition to the DC forms, which egress around 36 hours post infection (Troese and Carlyon, 2009). The *E. chaffeensis* developmental cycle is slightly longer, with DC to RC transition occurring over 24 hours and log growth of the RC for 48 hours prior to differentiation back to the DC at about 72 hours post infection (Zhang et al., 2007). *A. phagocytophilum* and *E. chaffeensis* T1SS and T4SS effector proteins modulate host cell processes which support pathogen vacuole maintenance and bacterial growth. While host cell rupture enables infection of nearby cells, *A. phagocytophilum* and *E. chaffeensis* also regulate host cell egress, with *A. phagocytophilum* utilizing exocytosis (Read et al., 2022) and *E. chaffeensis* hijacking filopodia to infect neighboring cells (Thomas et al., 2010).

### Chlamydia trachomatis

Transmission and cell tropism of *Chlamydia* spp. varies depending on the species and serovar, with *C. trachomatis* primarily infecting epithelial cells. *C. trachomatis* is a significant cause of sexually transmitted disease and trachoma worldwide. *C. trachomatis* entry into host cells involves initial low-affinity interactions between host heparan sulfate proteoglycans (HSPGs) and bacterial outer membrane complex protein (OmcB) and major outer membrane protein (MOMP) (Zhang and Stephens, 1992; Su et al., 1996; Fadel and Eley, 2008). This initial interaction, followed by binding to multiple host receptors such as fibroblast growth factor receptor (FGFR), ephrin A2 receptor (EPHA2), and epithelial growth factor receptor (EGFR), triggers signaling pathways that lead to actin reorganization around the attached bacteria (Kim et al., 2011; Patel et al., 2014; Subbarayal et al., 2015). Subsequently, membrane invagination, facilitated by clathrin or caveolae-mediated processes, initiates the entry of *C. trachomatis* into the host cell (Gabel et al., 2004; Webley et al., 2004; Majeed and Kihlström, 1991). Following host cell attachment and internalization, the bacteria block phagosome maturation and reside in a membrane-bound compartment known as the inclusion. While non-fusogenic with endosomes and lysosomes, the *C. trachomatis* inclusion intercepts Golgi-derived vesicles and multivesicular bodies (Hackstadt et al., 1995; Hackstadt et al., 1996; Heinzen et al., 1996; Beatty, 2006) which deliver nutrients as well as membrane to support inclusion expansion. *C. trachomatis* T3SS effector proteins are necessary for entry, inclusion formation and maintenance, and nutrient acquisition. The biphasic developmental cycle of *C. trachomatis* consists of an infectious non-replicative elementary body (EB) and the non-infectious replicative reticulate body (RB). While the timing of differentiation varies between species and serovars, EBs differentiate into RBs, which replicate and then undergo secondary differentiation to form new EBs prior to host cell egress and reinfection. *C. trachomatis* egress can occur by one of two mechanisms: an active extrusion process or cell death *via* lysis (Lutter et al., 2013).

### Coxiella burnetii

*Coxiella burnetii* causes human Q Fever, an aerosol-borne zoonotic disease typically transmitted from sheep and goats. *C. burnetii* exists in two morphological forms: the small cell variant (SCV) is metabolically inactive and environmentally stable, while the large cell variant (LCV) is metabolically active but cannot survive long term outside of the host cell. Unlike the *C. trachomatis* developmental forms, both SCVs and LCVs are capable of infecting cells through phagocytosis, though infection typically begins with inhalation of SCV-contaminated aerosols. In the lung, *C. burnetii* displays tropism for alveolar macrophages but will infect a wide range of both professional and non-professional phagocytic cells. *C. burnetii* host cell entry occurs through a passive mechanism involving αvβ3 integrins and subsequent reorganization of the actin cytoskeleton (Meconi et al., 1998; Capo et al., 1999; Rosales et al., 2012; Gilk et al., 2013). Upon host cell uptake, the *C. burnetii*-containing phagosome matures through the endocytic pathway to a phagolysosome (Howe and Mallavia, 2000; Howe et al., 2003), with the acidic pH of the phagolysosome activating *C. burnetii* metabolism and differentiation to the LCV (Hackstadt and Williams, 1981). SCV to LCV maturation begins almost immediately, with many of the bacteria transitioning by 2 hours post infection (Howe and Mallavia, 2000). In the first 24 hours of infection the bacteria undergo one or two rounds of replication, with the vacuole remaining small and tightfitting (Justis et al., 2017). Between 24- and 48-hours post-infection the vacuole rapidly expands, presumably through fusion between the *C. burnetii-*containing vacuole (CCV) and host endosomes, lysosomes, and autophagosomes. T4SS effector proteins are essential for forming and maintaining the mature CCV, a large, moderately acidic (pH ~5.2) vacuole that supports the log phase of bacterial replication, with a doubling time of 10-12 hours (Beare et al., 2009). LCV transition to SCV occurs around 4-6 days post-infection, though this may be cell-type dependent (Coleman et al., 2004). *C. burnetii* does not appear to have an active egress mechanism, but eventual host cell death releases bacteria to infect neighboring cells and repeat the growth cycle.

## Trafficking of the nascent pathogen-containing vacuole

**Figure 1:**
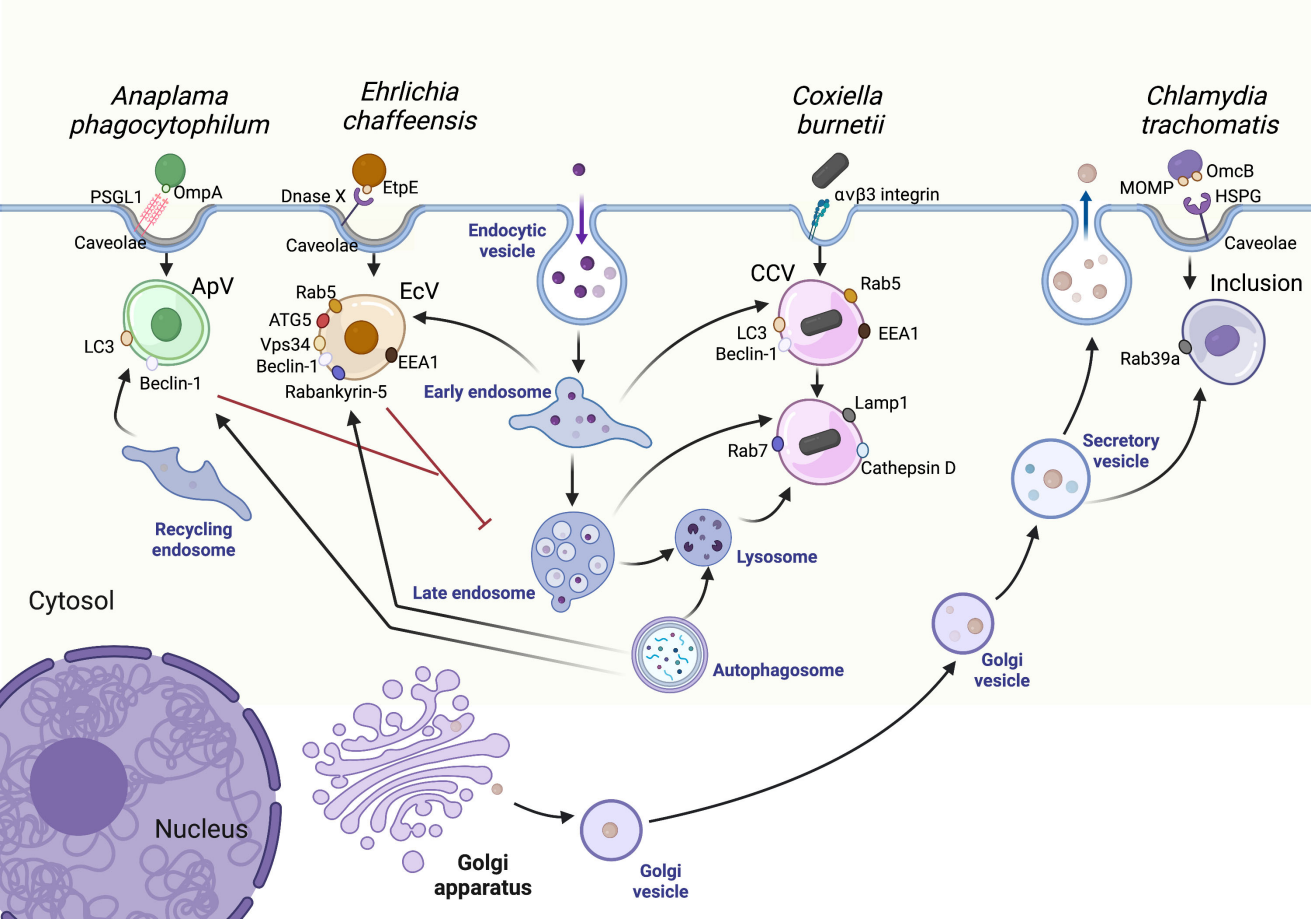
Pathogen interactions with the host vesicular trafficking pathways. Vesicular trafficking pathways in the host cell are rich in amino acids, lipids, and other resources essential for pathogen replication. The *Ehrlichia chaffeensis* vacuole (EcV) interacts with early endosomes and autophagosomes while the *Anaplasma phagocytophilum* vacuole (ApV) primarily interacts with autophagosomes and recycling endosomes. The *Chlamydia trachomatis* inclusion intercepts secretory and other Golgi-derived vesicles. Finally, *Coxiella burnetii* survives in a vacuole known as the CCV, a modified phagolysosome which readily interacts with host endosomes and autophagosomes. CCV, *Coxiella* containing vacuole; ApV, *Anaplasma phagocytophilum* containing vacuole; EcV, *Ehrlichia chaffeensis* containing vacuole. Created with Biorender.

A bacterium-containing phagosome typically proceeds through the endosomal maturation pathway, delivering the bacterium to a mature phagolysosome for degradation. Phagosome maturation begins through interactions with the early endosome, a pleomorphic, moderately acidic (pH 6.1 – 6.8) vesicle which receives and sorts internalized cargo (Helenius et al., 1983). Early endosomes fuse with Golgi-derived vesicles carrying newly synthesized proteases and hydrolases to form late endosomes (pH 4.9 - 6.0) (Maxfield and Yamashiro, 1987). Late endosomes finally fuse with lysosomes, forming a phagolysosome which maintains a pH<4.7 to support proteolytic activity of cathepsins and other degradative enzymes (Huotari and Helenius, 2011). Thus, early endosomes, late endosomes, and lysosomes constitute a highly dynamic pathway that traffics and degrades cellular cargo, including bacteria (Scott et al., 2014). As most bacteria cannot survive the acidic pH and proteolytic activity of the phagolysosome, pathogens must manipulate the initial trafficking events of the nascent phagosome to establish the infection. Endosomal maturation is regulated in large part by small GTPase Ras-associated binding (Rab) proteins. Rab proteins switch between their active (GTP-bound) and inactive (GDP-bound) states with the assistance of guanine nucleotide exchange factors (GEFs) which catalyze the release of GDP to allow GTP binding and Rab activation, while GTPase-activating proteins (GAPs) stimulate GTP hydrolysis and thus Rab inactivation. Rab proteins localize to specific vesicular membranes and recruit additional proteins, such as SNARE fusion proteins, to mediate trafficking and fusion events. Given their highly specific localizations, Rab proteins serve as markers for subcellular compartments and progression of cellular processes, including endosomal maturation. For example, loss of Rab5 (early endosome) followed by acquisition of Rab7 (late endosome) is a key step in endosomal maturation. With the complex and highly regulated series of events that occur during endosomal maturation, bacterial pathogens have a plethora of proteins and processes to target in order to avoid delivery to a compartment incompatible with pathogen survival (**Figure 1**).

*C. burnetii* uniquely requires an acidic environment to activate bacterial metabolism (Hackstadt and Williams, 1981), with the nascent phagosome progressing through the endosomal maturation pathway to a phagolysosome. While Rab5 to Rab7 conversion indicates that the nascent *C. burnetii* phagosome converts from an early to late endosome with similar kinetics as beads (Romano et al., 2007), slower acid phosphatase acquisition suggests delayed phagosome-lysosome fusion (Howe and Mallavia, 2000). Moreover, delayed maturation of the phagosome appears to be a bacteria-driven process, as inhibiting *C. burnetii* protein synthesis accelerates localization of the lysosomal protease cathepsin D to the CCV (Romano et al., 2007). *C. burnetii* also induces interactions between the nascent bacterium-containing phagosome and the autophagic pathway, with autophagy supporting CCV formation (Gutierrez et al., 2005; Romano et al., 2007). Most *C. burnetii-*containing phagosomes are positive for the autophagy marker LC3 by 1 hour post infection (Romano et al., 2007; Schulze-Luehrmann et al., 2016) and inducing autophagy delays acquisition of cathepsin D and therefore fusion with lysosomes (Romano et al., 2007). Thus, interactions with the autophagic pathway during the initial stages of infection may stall phagosome maturation and allow the bacteria time to adapt to their new environment. Intriguing data suggest that fusion between the nascent phagolysosome and autophagosomes “repairs” phagosomes that are initially damaged (Mansilla Pareja et al., 2017), thus promoting *C. burnetii* infection. Significant questions remain as to whether *C. burnetii* T4SS effector protein(s) play a role in delaying phagosome maturation, as well as whether SCV and LCV-containing phagosomes have identical maturation kinetics.

*A. phagocytophilum*, and *E. chaffeensis*, unlike *C. burnetii*, actively avoid fusion between the nascent pathogen-containing vacuole and lysosomes. *E. chaffeensis* achieves this by blocking Rab5 to Rab7 conversion, thus stalling the EcV as an early endosome. The *E. chaffeensis* T4SS effector protein Etf-1 recruits Rab5-GTP, while Etf-2 competitively inhibits hydrolysis of Rab5-GTP by RabGap5, thus interfering with Rab5 to Rab7 conversion (Lin et al., 2016; Yan et al., 2018). Rab5 appears to be a key component of the EcV, with the Rab5 effector proteins EEA1, Vps34, and Rabankyrin-5 also found on the EcV (Barnewall et al., 1997; Lin et al., 2016). The EcV also fuses with autophagosomes, forming an amphisome with autophagy markers including LC3, Beclin-1, and p62 (Lin et al., 2016; Lina et al., 2017). In contrast, the *A. phagocytophilum* vacuole (ApV) does not mature along the endocytic pathway, as it does not acquire markers for early or late endosomes and lysosomes (Webster et al., 1998; Mott et al., 1999). While not fusing directly with multivesicular bodies (MVBs), the presence of intraluminal vesicles and lysobisphosphatidic acid (LBPA) suggest that the ApV has MVB characteristics (Read et al., 2022). The ApV harbors markers for recycling endosomes, with bacterial-dependent recruitment of Rab GTPases typical of recycling endosomes and those that mediate endoplasmic reticulum to Golgi trafficking (Huang et al., 2010a). Autophagosome also contribute to ApV formation, based on the presence of autophagy markers and the ApV double membrane (Niu et al., 2008). Interestingly, the ApV membrane is monoubiquitinated shortly after entry, which may serve to hide the ApV and avoid fusion with lysosomes (Huang et al., 2012).

While the CCV, ApV, and EcV resemble modified endosomal compartments, the *C. trachomatis* inclusion quickly dissociates from the endosomal pathway. *C. trachomatis* protein synthesis is required to quickly isolate the inclusion from the endosomal pathway, with a lack of markers for early endosomes, lysosomes, or recycling endosomes on the inclusion in the first two hours of infection (Scidmore et al., 1996; Scidmore et al., 2003). Around 2 hours post infection, however, tubular endosomes associate with the inclusion in a bacterial-dependent manner, although there is no clear fusion event and the function is not clear (Scidmore et al., 2003). Shortly after infection, *C. trachomatis* inclusion proteins, known as ‘Incs’, remodel the inclusion membrane and facilitate interactions with host organelles and vesicular trafficking, in particular secretory vesicles originating from the Golgi. *C. trachomatis* drives dynein-dependent trafficking of the inclusion along microtubules to the microtubule organizing center (MTOC), where the inclusion remains associated with centrosomes for the remainder of the infection (Higashi, 1965; Clausen et al., 1997; Grieshaber et al., 2003).

## Homotypic fusion

Homotypic fusion of pathogen-containing vacuoles, which occurs when multiple bacteria enter the same cell, has been described for *C. burnetii* CCVs and *C. trachomatis* inclusions, but not for *A. phagocytophilum* or *E. chaffeensis*. CCV homotypic fusion involves autophagy, with both bacterial and host proteins playing key roles. While not on the CCV, the host autophagosomal SNARE protein Syntaxin 17, which mediates autophagosome-lysosome fusion (Itakura and Mizushima, 2013), indirectly promotes and is required for CCV homotypic fusion (Mcdonough et al., 2013). Vamp7, a SNARE protein involved in late endosome-lysosome fusion, localizes to the CCV and increases CCV homotypic fusion when overexpressed (Campoy et al., 2013). The *C. burnetii* T4BSS effector protein CvpB/Cig2 stabilizes autophagic machinery by modulating phosphatidylinositol 3-phosphate PI(3)P metabolism, which is a key lipid on autophagosomes (Martinez et al., 2016). CvpB/Cig2 mutants grow normally in cells but are defective in homotypic fusion, indicating homotypic fusion is not essential for growth *in vitro* (Newton et al., 2014; Larson et al., 2015; Martinez et al., 2016). Interestingly, CvpB/Cig2 is required for growth in the virulence insect model *Galleria mellonella*, suggesting a link between CvpB/Cig2, autophagy, homotypic fusion, and pathogenesis (Martinez et al., 2016). However, the phenotype may be directly related to homotypic fusion, or another aspect of Cig2/CvpB-regulated PI(3)P metabolism. It is not clear whether Syntaxin 17, CvpB/Cig2, and Vamp7 function in the same pathway or interact with one another to regulate CCV homotypic fusion. Finally, while not required during an *in vitro* infection, it remains to be seen whether homotypic fusion is critical for *in vivo* mammalian infection.

*C. trachomatis* inclusion fusion requires bacterial protein synthesis (Van Ooij et al., 1998), indicating that the bacteria actively promote this process. During *C. trachomatis* infection, homotypic fusion involves the inclusion protein IncA, which is structurally and functionally similar to eukaryotic SNARE proteins (Richards et al., 2013; Cingolani et al., 2019). A natural mutant in IncA is defective in homotypic fusion, does not replicate well in humans and causes more mild disease (Suchland et al., 2000; Geisler et al., 2001; Pannekoek et al., 2005). However, a targeted IncA mutant is also defective in homotypic fusion but grows normally in cells *in vitro* (Weber et al., 2016), leaving IncA function in chlamydial pathogenesis unresolved. For both *C. trachomatis* and *C. burnetii*, the role of homotypic fusion *in vivo* is not clear, but may enable the bacteria to “pool their resources” and quickly subvert the host cell before an innate immune response can control the infection. In the case of *Chlamydia*, genetic exchange occurs following homotypic fusion of different *C. trachomatis* serovars (Ridderhof and Barnes, 1989), suggesting an evolutionary advantage to homotypic fusion.

## Membrane content of mature pathogen-containing vacuoles

Pathogen-containing vacuoles are primarily composed of host-derived membranes yet are substantially modified by the bacteria through insertion of bacterial proteins. In addition, because the bacteria selectively regulate interactions between the vacuole and host vesicles and organelles, each pathogen-containing vacuole has a unique lipid and protein content. For example, the *C. trachomatis* inclusion membrane has a unique lipid content consisting of cholesterol (Hackstadt et al., 1995; Hackstadt et al., 1996; Wylie et al., 1997; Carabeo et al., 2003), sphingomyelin (Hackstadt et al., 1996), diacylglycerol (Tse et al., 2005), and phosphoinositides (Moorhead et al., 2010). Sphingomyelin is especially important for the *C. trachomatis* inclusion, as it has been implicated in maintaining inclusion membrane integrity, bacterial differentiation, and inclusion homotypic fusion (Robertson et al., 2009). Host-derived membrane components, such as phosphatidylinositol 3-phosphate (PI3P), glycerophospholipids and cholesterol localize to the membrane of *E. chaffeensis*-containing vacuole (EcV) and are absolutely required for *E. chaffeensis* proliferation (Lin et al., 2016; Lin et al., 2020).

Cholesterol is an essential component of mammalian cells and highly enriched in plasma membrane regions known as lipid rafts or microdomains; further, cholesterol is found in the pathogen containing vacuoles for *A. phagocytophilum, E. chaffeensis, C. trachomatis, and C. burnetii* (Carabeo et al., 2003; Lin and Rikihisa, 2003a; Howe and Heinzen, 2006; Xiong et al., 2009). Cholesterol accumulation in ApVs supports *A. phagocytophilum* replication (Xiong et al., 2009), but the intracellular replication of *C. burnetii* and *C. trachomatis* is independent of cholesterol, as bacterial growth in cholesterol-free cells is unaffected (Gilk et al., 2013). Cholesterol-rich microdomains are present in the membranes of pathogen-containing vacuoles for *C. burnetii* (Howe and Heinzen, 2006), *C. trachomatis* (Carabeo et al., 2003; Mital et al., 2010) and *A. phagocytophilum* (Xiong et al., 2009). Of note, the membranes of both CCVs and ApVs contain the lipid raft proteins flotillin-1 and flotillin-2 (Howe and Heinzen, 2006; Xiong et al., 2019). While the function of cholesterol-rich microdomains in the CCV remains elusive, flotillin-1 and flotillin-2 are absolutely required for LDL-derived cholesterol trafficking to ApVs (Xiong et al., 2019). In the *C. trachomatis* inclusion, these microdomains co-localize with a subset of bacterial inclusion membrane proteins (Incs), including IncB, IncC, CT101, CT222, CT223, CT224, CT228, CT288, and CT850, and active Src-family kinases (Mital et al., 2010). Importantly, these microdomains are implicated in the interactions of the *C. trachomatis* inclusion with the microtubule network and centrosomes (Mital et al., 2010). Although Src-family kinases are not required for Inc microdomain formation, *C. trachomatis* species that lack active Src-family kinase-enriched microdomains on their inclusion membrane, like *C. muridarum* and *C. caviae*, do not traffic to the microtubule organizing center (MTOC), an important step during the *C. trachomatis* intracellular developmental cycle (Mital and Hackstadt, 2011). The Ca^2+^ sensor stromal interaction molecule 1 (STIM1) and the Ca^2+^ channel inositol-1,4,5-trisphosphate receptor, type 3 (ITPR3) also localize to active Src-family-kinase rich microdomains on the inclusion membrane, and their interaction with the inclusion membrane protein CT101, or Myosin Regulatory Complex subunit A (MrcA), promotes extrusion of the inclusion at the end of *C. trachomatis’s* intracellular developmental cycle (Nguyen et al., 2018). In addition, CT228 recruits elements of the myosin phosphatase pathway, and also plays a role in *C. trachomatis* extrusion (Lutter et al., 2013). The precise function of all *C. trachomatis* proteins identified in microdomains has not yet been determined but elucidating how these multiple proteins interact will substantially contribute to a better understanding of *C. trachomatis’s* pathogenesis.

The *C. trachomatis* inclusion proteins (Incs) are secreted through the T3SS and then inserted into the inclusion membrane. These unique proteins consist of at least two transmembrane domains with a short linker, with both the N- and C-termini on the host cytosolic side of the inclusion (Bannantine et al., 1998; Scidmore-Carlson et al., 1999; Lutter et al., 2012). *C. trachomatis* expresses more than 50 Inc proteins at different times during the developmental cycle, suggesting that Incs play specific roles during infection (Shaw et al., 2000; Gauliard et al., 2015; Rucks et al., 2017). The *C. burnetii* vacuolar proteins (Cvps) are a family of six effector proteins translocated through the T4BSS Dot/Icm secretion system which localize to the vacuolar membrane (Larson et al., 2013; Larson et al., 2015). Bacteria mutants lacking cvpA, cvpB, cvpC, cvpD, cvpE and cvpF exhibit defects in intracellular growth and CCV biogenesis, indicating that these Cvps are required to promote *C. burnetii* replication (Larson et al., 2013; Newton et al., 2014; Larson et al., 2015; Martinez et al., 2016; Siadous et al., 2021). Finally, the ApV is actively modified by the bacterial protein APH_1387, which localizes to the ApV membrane and shares secondary structure characteristics with *C. trachomatis* Inc proteins (Huang et al., 2010b).

## Microenvironment of pathogen containing vacuoles

Successful pathogens evade host immune defenses by remodeling their intracellular niches into safe compartments. While the membrane serves as the interface with the host cell, the luminal environment supports bacterial replication. The pH of pathogen-containing compartments is a critical feature, given the effect not only on the enzymatic activity of luminal proteins (e.g., cathepsins in the CCV), but also on the metabolic activity of the bacteria. This is perhaps most evident for *C. burnetii*, which resides in a modified phagosome. *C. burnetii* metabolic activity is initially activated by the acidic pH (pH < 4.7) of the phagolysosome (Hackstadt and Williams, 1981), while the modified CCV pH elevates to an average pH of 5.2 by 1 day post infection (Samanta et al., 2019). Intriguingly, cathepsins and other lysosomal proteases in the CCV lumen do not affect *C. burnetii*, yet will degrade other bacteria such as *E. coli* (Howe et al., 2010). Intriguingly, cholesterol accumulation on the CCV membrane acidifies the CCV to pH~4.8, which leads to *C. burnetii* degradation (Mulye et al., 2017). The mechanism for *C. burnetii* degradation in acidified CCVs requires further investigation, but is likely not directly mediated by the pH, as the optimal pH for *C. burnetii* in axenic cultures is 4.5-4.75 (Vallejo Esquerra et al., 2017). Presumably, *C. burnetii* death in acidified CCVs is linked to the increased proteolytic activity of lysosomal degradative enzymes, beyond the threshold tolerated by the bacteria. Indeed, we recently demonstrated that in order to build a permissive intracellular niche, *C. burnetii* actively regulates CCV acidity by inhibiting endosomal maturation, further reducing the number of proteolytically active lysosomes available for heterotypic fusion with the CCV (Samanta et al., 2019). It is possible that *C. burnetii* utilizes additional mechanisms to maintain optimal CCV pH, such as secreting neutralizing enzymes into the CCV lumen or blocking endosomal proton pumps. Elucidating how *C. burnetii* regulates CCV pH is an important topic for further study, given its critical role in bacterial intracellular growth and pathogenesis.

Given that *C. trachomatis* avoids lysosomal fusion, it is not surprising that the *C. trachomatis* inclusion has a more neutral pH (pH:7.25) (Grieshaber et al., 2002). Preliminary experiments suggest the *A. phagocytophilum* vacuole (ApV) and *E. chaffeensis* containing vacuole (EcV) pH are ~5.2 using LysoSensor Green DND-189, a dye that becomes more fluorescent in acidic environments (Cheng et al., 2014a; Read et al., 2022). Interestingly, it was first demonstrated that the axenic medium pH does not have a significant impact on protein biosynthesis of *E. chaffeensis* reticulate cells (RCs) (Eedunuri et al., 2018). However, a recent study found that the levels of protein and DNA synthesis in axenic medium of host cell-free phagosomes containing *E. chaffeensis* and *A. phagocytophilum* are the highest at neutral pH (Zhang et al., 2021), which may be due to other factors related to the presence of phagosomes, supporting the hypothesis that an acidic pH within the vacuoles indirectly affects bacterial survival.

## Interactions of the mature pathogen containing vacuole with host trafficking pathways

Sheltering in a membrane-bound vacuole presents certain challenges, particularly for pathogens such as *A. phagocytophilum* and *C. trachomatis* which have reduced genomes and rely on the host for key nutrients. These pathogens utilize sophisticated mechanisms to hijack host amino acids, lipids, and other essential resources. One common approach is diverting host vesicular trafficking to the pathogen-containing vacuole, with fusion events delivering nutrients to the vacuole lumen for bacterial uptake. Vesicular fusion also directly provides membrane for the expanding pathogen-containing vacuole. The common pathways targeted involved endosomal trafficking, secretion, and autophagy (**Figure 1**).

## Heterotypic fusion with the endocytic pathway

As described earlier, the endocytic pathway delivers extracellular cargo to lysosomes for degradation. Early endosomes, which form through homotypic fusion of endocytic vesicles or heterotypic fusion between endocytic vesicles and early endosomes, concentrate newly internalized cargo for sorting (Salzman and Maxfield, 1989). Early endosomes are dynamic and morphologically pleomorphic, forming tubules which concentrate receptors while released cargo is concentrated in the endosome lumen. From the early endosome, certain receptors (e.g., transferrin receptor and low density lipoprotein receptors) are recycled back to the plasma membrane in recycling endosomes, while the majority of cargo is delivered *via* late endosomes to lysosomes for degradation. A specialized subset of late endosomes known as multivesicular bodies (MVB) contain internal vesicles that form by inward budding of cytosolic contents and are cholesterol-rich. Given the nutrient-rich contents of vesicles within the endocytic pathway, re-routing host endosomes to pathogen-containing vacuoles provides access to essential nutrients such as iron and cholesterol. For those pathogens that interact with lysosomes, the lysosomal degradative capacity also provides a source of peptides and amino acids to support bacterial growth.

Based on markers, the mature vacuoles harboring *C. burnetii, C. trachomatis*, and *A. phagocytophilum* do not interact with early endosomes (Barnewall et al., 1997; Barnewall et al., 1999; Mott et al., 1999; Huang et al., 2010a). However, the mature EcV has characteristics of an early endosome, including the presence of early endosomal markers Rab5, transferrin receptor, EEA1, Vps34, and Rabankyrin-5 (Barnewall et al., 1997; Rikihisa, 2015; Lin et al., 2016), and does not fuse with late endosomes and lysosomes (Barnewall et al., 1997). EcV heterotypic fusion with early endosomes and recycling endosomes delivers iron to the bacteria, with the mature EcV accumulating transferrin receptor (Barnewall et al., 1997). *E. chaffeensis* actively subverts transferrin trafficking, based on the lack of transferrin on the early EcV and pathogen-induced upregulation of transferrin receptor expression (Barnewall et al., 1997; Lin and Rikihisa, 2003b). In addition to iron, early endosomal fusion supplies membrane for both the bacteria and expanding vacuole (Lin et al., 2020). While the bacterial-driven mechanisms for EcV fusion with the endocytic pathway are poorly understood, the *E. chaffeensis* effector protein Etf-1 induces fusion between autophagosomes and multivesicular bodies (MVBs) to form amphisomes, which then deliver cargo-containing intraluminal vesicles to the EcV lumen (Lin et al., 2016; Lin et al., 2020). Given that MVBs are cholesterol-rich, they may provide lipids and membrane necessary for bacterial replication.

While the ApV does not interact with early and late endosomes or lysosomes, the bacteria actively recruit a special subset of cholesterol-rich endosomal recycling endosomes to provide cholesterol for the bacteria (Lin and Rikihisa, 2003b; Huang et al., 2010a). These vesicles are rich in low density lipoprotein (LDL)-derived cholesterol; *A. phagocytophilum* upregulates LDL receptor expression, leading to increased endocytosis of LDL-derived cholesterol to support bacterial replication (Xiong et al., 2009). Cholesterol-enriched vesicles positive for the lipid raft proteins flotillin 1 and flotillin 2, as well as the cholesterol transporter NPC1, traffic to the ApV (Xiong et al., 2019). Intriguingly, intraluminal membranes containing NPC1 and flotillin 2 surround the bacteria inside the ApV, a process which requires *A. phagocytophilum* protein synthesis, indicating this is a bacterial-driven process (Huang et al., 2021). While the bacterial proteins involved in recruitment of these vesicles are unknown, the Golgi-derived SNARE proteins VAMP4 and STX16, which are both involved in LDL vesicular transport, localize to the ApV (Xiong and Rikihisa, 2012). Further, VAMP4 is required for *A. phagocytophilum* infection, leading to the hypothesis that *A. phagocytophilum* subverts VAMP4 and STX16 to selectively recruit NPC1-positive vesicles rich in LDL-cholesterol (Xiong and Rikihisa, 2012).

During *C. burnetii* infection, the mature CCV does not associate with early endosomes, based on the lack of markers such as EEA1 and Rab5 (Howe et al., 2003; Romano et al., 2007). However, heterotypic fusion with late endosomes, MVBs, and lysosomes readily occurs at both early and late stages of infection, most likely serving as a source of membrane and nutrients (Heinzen et al., 1996; Clemente et al., 2022). CCV-lysosome fusion may provide amino acids needed to support *C. burnetii* growth and activate the T4BSS, particularly at the very early stages of infection (Newton et al., 2020). While the fact that *C. burnetii* is auxotrophic for multiple amino acids supports this hypothesis (Sandoz et al., 2016), other studies have shown that *C. burnetii* growth and CCV formation does not require lysosomal hydrolases, and therefor lysosomal degradation of proteins, for growth (Miller et al., 2019). Further, *C. burnetii* blocks endosomal maturation as early as one day post infection, leading to decreased host lysosomes and expansion of the late endosomal population (Larson and Heinzen, 2017; Samanta et al., 2019). While the fusion rate is similar between 2 days post infection (prior to expansion) and 3 days post infection (after expansion) (Samanta et al., 2019; Clemente et al., 2022), it is likely that different endosomal populations fuse with the CCV at different points during infection. At the molecular level, several host SNARE proteins localize to the CCV and play an important role in CCV expansion through heterotypic fusion. VAMP8, which is involved in homotypic fusion between endosomes, is recruited to early CCVs but is not present on the mature CCV (Campoy et al., 2013). Conversely, VAMP3 is present only on mature CCVs and facilitates CCV fusion with MVBs (Campoy et al., 2013). VAMP7 and Vti1b, which are involved in heterotypic fusion between late endosomes and lysosomes, are both actively recruited to and present on the mature CCV, with VAMP7 being essential for CCV expansion (Campoy et al., 2013). Actin patches on the CCV, while not required, do promote clustering of the SNARE proteins VAMP7 and syntaxin 8 on the CCV for localized fusion (Miller et al., 2018). Rab7 and the HOPS tethering complex both localize to the CCV and promote vesicular fusion (Barry et al., 2012). Intriguingly, Synaptotagmin VII, a calcium sensor for membrane fusion, as well as the cAMP-regulated guanine nucleotide exchange factor EPAC and the RAG GTPase Rap1, localize to the CCV and may regulate CCV fusion with the host endosomal pathway (Campoy et al., 2013; Mansilla Pareja et al., 2017). Thus, while critical for CCV formation and maintenance, the molecular mechanisms underlying CCV fusion with the endosomal pathway is poorly understood.

The *C. trachomatis* inclusion does not directly fuse with endosomal vesicles (Heinzen et al., 1996; Rzomp et al., 2006), with the exception of MVBs (Beatty, 2006). MVBs are enriched for sphingolipids and cholesterol, both of which are necessary for intracellular *C. trachomatis* growth (Hackstadt et al., 1995; Hackstadt et al., 1996; Van Ooij et al., 2000; Carabeo et al., 2003). While cholesterol is not absolutely required, host cell sphingomyelin synthesis is essential for biogenesis of the *C. trachomatis* inclusion membrane and stability (Robertson et al., 2009; Gilk et al., 2013). *C. trachomatis* recruits Rab39a to the inclusion, where it specifically regulates trafficking of sphingomyelin and phospholipids from MVBs to the inclusion (Gambarte Tudela et al., 2015; Gambarte Tudela et al., 2019). While lysosomes provide nutrients necessary to support *C. trachomatis* growth (Ouellette et al., 2011), the *C. trachomatis* inclusion protein IncA actively blocks heterotypic fusion between the inclusion and host lysosomes. IncA contains a SNARE-like motif which acts as a competitive inhibitor of heterotypic membrane fusion between the inclusion and lysosomes, while promoting homotypic fusion between inclusions (Delevoye et al., 2008; Paumet et al., 2009; Ronzone et al., 2014).

## Secretory pathway

The host secretory pathway transports newly synthesized proteins and lipids from the ER to the Golgi, where they are sorted for delivery to various cellular locations including the plasma membrane and endosomes. At specialized ER exit sites, the Rab GTPase Sar1 recruits the vesicle coat protein COPII to form cargo-loaded vesicles which transit to the ER-Golgi intermediate compartment (ERGIC). Rab1 and Rab2 are then responsible for trafficking from the ERGIC to the cis-Golgi where proteins undergo progressive modification, primarily glycosylation, as they are transported to the trans-Golgi (TGN). The TGN serves as a sorting platform to package proteins and lipids for constitutive or regulated secretion at the plasma membrane, as well as transport to the endolysosomal system. Cathepsins and other lysosomal proteins are specifically tagged by mannose-6-phosphate (M6P) in the cis-Golgi and transported to late endosomes by the mannose-6-phosphate receptor (M6PR). In order to obtain lipids and membrane, intracellular pathogens commonly subvert the secretory pathway by targeting the key regulatory proteins M6PR, Rab and SNARE.

Interaction with Golgi-derived vesicles is a hallmark of the *C. trachomatis* inclusion, which is decorated with numerous Golgi proteins (Rzomp et al., 2003; Webley et al., 2004; Gambarte Tudela et al., 2019). Trans-Golgi derived vesicles destined for the plasma membrane are actively diverted to the *C. trachomatis* inclusion to deliver essential nutrients, such as sphingomyelin and cholesterol, which are incorporated into the bacterial membrane (Hackstadt et al., 1995; Hackstadt et al., 1996; Scidmore et al., 1996; Carabeo et al., 2003). Bacterial-induced Golgi fragmentation into mini-stacks around the inclusion occurs around 20 hours post infection; preventing Golgi fragmentation by blocking cleavage of the Golgi matrix protein golgin-84 negatively impacts C*. trachomatis* intracellular growth (Heuer et al., 2009). Two related secreted *C. trachomatis* effector proteins, ChlaDUB1 and ChlaDUB2, induce Golgi fragmentation through Lys63-deubiquitinase activity (Pruneda et al., 2018). *C. trachomatis* infection is enhanced by increasing Golgi complex ministack formation, presumably by improving access to nutrients in exocytic Golgi-derived vesicles (Heuer et al., 2009). *C. trachomatis* also directly targets the Golgi, secreting at least one protein, CteG, which localizes to the Golgi (Pais et al., 2019). In addition to secreted proteins, inclusion proteins recruit Golgi-derived vesicles. Inc protein CT813/InaC recruits ARF1 and ARF4 GTPases to the inclusion membrane, where they induce posttranslational modification of microtubules to stabilize Golgi ministacks around the inclusion (Wesolowski et al., 2017). Inc protein CT229/CpoS binds numerous Rab proteins involved in Golgi trafficking, diverting vesicular trafficking to the inclusion (Rzomp et al., 2006; Mirrashidi et al., 2015; Sixt et al., 2017; Faris et al., 2019). These include active Rab 4 and Rab35, which divert transferrin-containing recycling endosomes to the inclusion, where they provide iron to the bacteria (Rzomp et al., 2006; Faris et al., 2019). CT229 binds and recruits to the inclusion Rab1 (anterograde transport), Rab 6 (retrograde transport), Rab 8, 10, 14, and 34 (Golgi-transport) (Faris et al., 2019). Subversion of Golgi-derived vesicles requires numerous other host cell factors, including Arf1, Rab14, Rab6A, GBF1, STX10, and Rab11A (Rzomp et al., 2003; Rzomp et al., 2006; Heuer et al., 2009; Elwell et al., 2011; Lucas et al., 2015).

While there is no evidence that secretory vesicles are involved in *E. chaffeensis* infection, both *C. burnetii* and *A. phagocytophilum* selectively interact with the secretory pathway. Rab1, which regulates transport between the ER and the Golgi, is found on the CCV as early as 24 hours post-infection and is necessary for CCV expansion and downstream fusion with the endocytic pathway (Campoy et al., 2011). Rab1 may function in recruiting secretory vesicles to the CCV, which would then provide a source of membrane or nutrients necessary for CCV expansion and bacterial growth. One intriguing possibility is that early secretory vesicles deliver SNARE proteins, which then facilitate fusion with host endosomes and contribute with membrane to expanding CCV (Campoy et al., 2011). *A. phagocytophilum* also selectively interacts with the secretory pathway by recruiting Rab10-positive trans-Golgi-derived vesicles to the ApV lumen (Huang et al., 2010a; Truchan et al., 2016b). The *A. phagocytophilum* surface protein UMPK, a uridine monophosphate kinase, is a Rab10 ligand which may mediate interactions between the bacteria and Golgi-derived vesicles imported into the ApV lumen (Truchan et al., 2016b). These vesicles deliver sphingomyelin, which is not only incorporated into the bacterial membrane but also serve a signal for RC to DC transition and production of infectious bacteria (Truchan et al., 2016b). Like *C. trachomatis*, the Golgi fragments surround the ApV as bacterial load increases (Truchan et al., 2016b), suggesting the Golgi and secretory pathway may play a larger role during *A. phagocytophilum* infection than currently appreciated.

## Retromere and retrograde trafficking

Retrograde trafficking transports proteins and lipids from endosomes to the Golgi or plasma membrane, as well as from the Golgi to the ER. It is critical for protein recycling as well as delivering mis-delivered proteins to their correct location. The retromer complex, a trimer of Vps26, Vps29, and Vps35, regulates trafficking from Rab5/Rab7 endosomes to the trans-Golgi network and the plasma membrane (Personnic et al., 2016). The Vps complex binds Rab7 as well as sorting nexins (SNX1 or SNX2 and SNX5 or SNX6) on endosomes, which induce membrane curvature and endosome tubulation. Retrograde trafficking from late endosomes to the trans-Golgi network is essential for returning mannose-6-phosphate receptor (M6PR) back to the Golgi after the M6PR delivers lysosomal proteases to endosomes. Finally, retrograde transport within the Golgi stack and to the ER requires the COP1 coat protein, which is recruited by the small GTPase ARF1; other proteins involved include PtdIns 4-kinase IIIb, OCRL, and Rab6A (Personnic et al., 2016).

*C. trachomatis* actively disrupts retrograde trafficking of M6PR and SNX5 depletion enhances bacterial growth without affecting inclusion size, suggesting that the retromer restricts *C. trachomatis* infection (Aeberhard et al., 2015; Mirrashidi et al., 2015). Both SNX5 and SNX6 re-localize from endosomes to the *C. trachomatis* inclusion membrane, where they induce inclusion tubulation (Aeberhard et al., 2015; Mirrashidi et al., 2015). The *C. trachomatis* inclusion protein IncE binds to SNX5 and SNX6, disrupting binding between SNX5 and M6PR and trafficking of M6PR back to the Golgi (Mirrashidi et al., 2015; Elwell et al., 2017). Given that M6PR is critical for delivering newly synthesized hydrolases from the Golgi to endosomes, IncE disruption of the retromer and M6PR recycling may interfere with the degradative capabilities of lysosomes, which in turn benefits the bacteria. While retrograde trafficking from endosomes restricts *C. trachomatis* growth, retrograde trafficking within the Golgi supports *C. trachomatis* infection. The COG complex mediates intra-Golgi trafficking, functioning as membrane tethers and interacting with SNARES, Rabs, and COP1 proteins in the Golgi. Both COG subunits and COG-interacting proteins (e.g., Rab1, Rab6, Rab14, and syntaxin6), localize to the inclusion (Rzomp et al., 2003; Rejman Lipinski et al., 2009; Capmany et al., 2011; Moore et al., 2011). The Golgi SNARE protein GS15 also localizes to the inclusion in a COG-dependent manner, and depletion of Rab6, COG subunits and GS15 decreased growth. This indicates that *C. trachomatis* targets intra-Golgi trafficking, diverting these sphingomyelin-rich vesicles to the inclusion (Pokrovskaya et al., 2012).

There is no evidence that *A. phagocytophilum* or *E. chaffeensis* interact with retrograde trafficking pathway, and the role of retrograde trafficking during *C. burnetii* infection is not clear. One study found that depleting retromer components (VPS29, VPS35, SNX2, -3, and -5) inhibited *C. burnetii* growth, suggesting that retrograde trafficking benefits *C. burnetii* (Mcdonough et al., 2013). However, other studies indicate that Vps35 and Vps29 depletion, as well as chemical inhibitors of retrograde trafficking, have no effect on *C. burnetii* growth or CCV formation (Miller et al., 2018). Further, while *C. burnetii* does not appear to disrupt retrograde trafficking based on M6PR trafficking, retromer distribution is altered based on increased levels of VPS35 on late endosomes and lysosomes (Miller et al., 2018). Intriguingly, retromer components may play a role in fusion between the CCV and host endosomes, with VPS35 localizing to CCV actin patches which serve as fusion sites (Miller et al., 2018). One proposed model is that CCV Rab7 recruits retromer to the CCV, followed by formation of actin patches and HOPs/SNARE complexes mediating fusion between the CCV and late endosomes (Miller et al., 2018).

## Autophagy

Autophagy is a critical cellular process which eliminates damaged organelles and protein aggregates and enables recovery of nutrients. During macroautophagy, eukaryotic cells recycle damaged organelles, misfolded proteins, and other cargo by enclosing them in double membraned autophagosomes; fusion with lysosomes forms a degradative autolysosome. Lysosomes also directly participate in autophagy either through non-specific uptake of cytoplasmic components (microautophagy) or specifically engulfment of chaperone-bound cytosolic proteins (chaperone-mediate). During xenophagy, the autophagy machinery specifically targets cytosolic pathogens or the pathogen-containing vacuole, with the initial step being ubiquitination of the bacterial membrane or PCV membrane. During macroautophagy, the nascent autophagosome, or phagophore, forms at the ER, where the ULK1 complex (ULK1, ATG13, FIP200, and ATG101) recruits the phosphatidylinositol 3-kinase (PI3K) complex, including VPS34, p150, and Beclin-1 (Majeed et al., 2022). PI3K complex production of phosphatidylinositol 3-phosphate (PI3P) leads to recruitment of ATG9 and formation of the ATG12-ATG5-ATG16L1 complex, which then conjugates phosphatidylethanolamine (PE) to LC3 to form LC3-II, a hallmark of autophagosomes (reviewed in (Melia et al., 2020; Chang et al., 2021). Autophagosomes are specifically targeted to and fuse with endosomes and lysosomes through Rab GTPases and SNAREs. Autophagy can be induced by cellular stress through mTOR signaling, with active mTOR promoting biosynthesis pathways and inhibiting autophagy. Under conditions of cellular stress, including nutrient limitation, mTOR is inactivated, leading to upregulated expression of lysosomal and autophagosomal genes. While autophagy is a cellular innate immune response which can eliminate intracellular pathogens, many pathogens subvert autophagy to gain access to nutrients.

NP52, LC3, Beclin-1, and p62 are found on the CCV membrane, indicating that *C. burnetii* interacts with the autophagosomal pathway (Beron et al., 2002; Gutierrez et al., 2005; Romano et al., 2007; Vazquez and Colombo, 2010; Newton et al., 2013; Newton et al., 2014; Winchell et al., 2014; Kohler et al., 2016; Winchell et al., 2018; Dragan et al., 2019). While autophagy is not required for *C. burnetii* growth, autophagosomes likely contribute to CCV expansion both by directly fusing as well as contributing to homotypic fusion (Beron et al., 2002; Gutierrez et al., 2005; Mcdonough et al., 2013; Newton et al., 2014; Latomanski and Newton, 2018; Larson et al., 2019). LC3 and NDP52 presence in the CCV lumen indicates that autophagosomes directly fuse with the CCV, although direct conjugation of LC3 on the CCV membrane cannot be ruled out (Kohler et al., 2016; Mansilla Pareja et al., 2017). Xenophagy, which is selective autophagy of intracellular bacteria, does not play a significant role during infection (Lau et al., 2022). While p62 is recruited to the CCV in a T4BSS-dependent manner (Newton et al., 2014), the role of p62 appears to be primarily through signaling during oxidative stress rather than autophagy (Winchell et al., 2018). Several studies indicate that *C. burnetii* does not actively manipulate autophagic flux in epithelial cells and macrophages (Winchell et al., 2014; Winchell et al., 2018; Larson et al., 2019), but does so in neutrophils (Kumaresan et al., 2022). Numerous T4BSS effectors have been identified which interact with the autophagic pathway. CvpA and Cig57 both bind to components of clathrin-coated vesicles, thus facilitating fusion between autophagosomes and the CCV as well as CCV homotypic fusion (Larson et al., 2013; Latomanski et al., 2016; Latomanski and Newton, 2018). Indeed, CCV-localized clathrin heavy chain is essential for CCV expansion mediated by CCV-autophagosome fusion (Larson et al., 2013; Latomanski and Newton, 2018). CvpF recruits LC3B to the CCV and stimulates conversion to LC3B-II in a process tied to Rab26 activity (Siadous et al., 2021). CpeB traffics to the CCV and autophagosome-derived vesicles and co-localizes with LC3B (Voth et al., 2011), while CBU0513 is required for recruitment of lipidated LC3-II to the CCV (Crabill et al., 2018). CvpB/Cig2 mediates homotypic fusion and CCV-autophagosome fusion (Martinez et al., 2014; Newton et al., 2014; Larson et al., 2015; Kohler et al., 2016; Martinez et al., 2016). Mechanistically, CvpB interacts with PI3P and phosphatidylserine (PS) as mediated by its N-terminal region, and prevents the activity of the PI3P-5-kinase PIKfyve, thus blocking PI3P phosphorylation to PI(3,5)P2 (Martinez et al., 2016). This inhibition leads to the increase of PI3P at the CCV and favors vacuole fusion and LC3 recruitment (Martinez et al., 2016). Autophagy appears to repair damaged CCV membranes (Mansilla Pareja et al., 2017), while data suggests the ESCRT complex is involved in CCV membrane repair (Radulovic et al., 2018). *C. burnetii* inhibits mTOR in a non-canonical manner and without affecting autophagic flux (Larson et al., 2019). Finally, a potential link between autophagy and disease was observed in a study of genetic polymorphisms linked to chronic Q fever, where SNPs in ATG5 and MAP1LC3A were more commonly associated with controls, suggesting that autophagy promotes infection or more severe disease (Jansen et al., 2019).

The *C. trachomatis* inclusion does not directly fuse with autophagosomes (Al-Younes et al., 2004), and autophagy does restrict *C. trachomatis* growth in both epithelial cells and macrophages (Al-Younes et al., 2011; Yasir et al., 2011; Sun et al., 2012; Wang et al., 2021). *C. trachomatis* interferes with host ubiquitination of the inclusion by secreting a deubiquitinase Cdu1 (Haldar et al., 2016; Auer et al., 2020). In the absence of Cdu1, ubiquitination of the inclusion leads to recruitment of autophagy machinery and formation of a LC3 positive double membrane around the inclusion (Auer et al., 2020). However, it does not appear that this directly leads to bacterial clearance, but rather a growth defect due to inefficient recruitment of Golgi-derived vesicles necessary to support bacterial growth (Auer et al., 2020). This highlights the importance of the inclusion membrane in vesicular fusion. Curiously, *C. trachomatis* subverts the non-autophagy function of two host proteins, LC3 and ATG16L1. While LC3 does accumulate around the inclusion, this is not linked to autophagy but rather through interactions with the host protein MAP (microtubule-associate protein) which may serve to stabilize the microtubule network around the inclusion (Al-Younes et al., 2011). The *C. trachomatis* secreted effector protein CT622/TaiP binds to the host autophagy protein ATG16L1, blocking ATG16L1 interactions with the Golgi/late endosomal protein TMEM59 to reroute vesicular trafficking to the inclusion (Sadeh and Clopath, 2020). There is evidence that *C. trachomatis* induces autophagy in an ATG5-dependent manner at mid to late stages of development (Pachikara et al., 2009) and through p62 at later times during infection (Wang et al., 2021).

The ApV has several hallmarks of an autophagosome, including a double membrane and the presence of LC3 and Beclin 1 (Niu et al., 2008). Autophagy not only benefits *A. phagocytophilum* but also appears to be critical for growth (Niu et al., 2008; Niu et al., 2012). Like *C. trachomatis*, the ApV is mono-ubiquitinated (which can promote autophagy) in a process that requires bacterial protein synthesis (Huang et al., 2012). The *A. phagocytophilum* T4SS effector protein Ats-1 binds Beclin 1 to initiate autophagosome formation at the host ER (Niu et al., 2012). Autophagosomes appear to fuse with the ApV, delivering the contents to the ApV lumen (Niu et al., 2012). A second Ap T4SS effector protein, AptA, interacts with the proteosome protein PSMG3, activating the host ubiquitin-proteosome system to upregulate autophagy and support bacteria replication (Ma et al., 2021).

While the EcV is early endosome-like, autophagosome formation is required for *E. chaffeensis* replication (Lin et al., 2016). The autophagy markers ATG5 and Beclin 1 are found on the EcV (Bento et al., 2016; Lin et al., 2016), and localization of LC3 has been observed in some studies (Lina et al., 2017; Yan et al., 2018). Further, autophagosomes fuse with the EcV, delivering essential amino acids, especially glutamine, which is a primary energy and carbon source for *E. chaffeensis* (Cheng et al., 2014b; Lin et al., 2016). *E. chaffeensis* uses a different strategy than *A. phagocytophilum* to induce autophagy, which is independent of mTOR and ubiquitination pathways (Lin et al., 2016). Unlike *C. trachomatis* or *A. phagocytophilum*, the EcV is not ubiquitinated (Lin et al., 2016). The *E. chaffeensis* T4SS effector Etf-1 interacts with the PI3K complex, including Beclin 1, to recruit autophagosomes to the EcV as well as induce autophagy (Lin et al., 2016). Rab5 is also a key player in EcV autophagy; it appears that Rab5 is locked in the GTP-bound state on the EcV, which stabilizes interactions with the PIK3C3 (class III PtdIns3K) complex required to recruit Beclin 1 (Lin et al., 2016). Curiously, *E. chaffeensis* induces autophagosome formation but inhibits fusion between autophagosomes and lysosomes, thus downregulating autophagic degradation (Lina et al., 2017). The *E. chaffeensis* T1SS Trp effectors TRP120, TRP32, and TRP47 activate Wnt and PI3K/Akt signaling pathways to downregulate mTOR signaling to decrease autolysosome formation by decreasing TFEB translocation (Lina et al., 2017).

## Vacuole interactions with host organelles

While subverting vesicular trafficking is a productive source of nutrients, intracellular pathogens also directly target host organelles through membrane contact sites (MCS). MCS are locations where two or more membranes are within an intermembrane distance of less than 30nm, allowing for lipid and small molecule (e.g., calcium) exchange (Scorrano et al., 2019). In eukaryotic cells, inter-organelle MCS with the endoplasmic reticulum are common, given the essential roles the ER plays in lipid and protein synthesis, calcium storage and the cellular stress response. MCS formation and function relies on multi-protein complexes that tether the two membranes as well as physically transfer lipids or other small molecules. Both *C. trachomatis* and *C. burnetii* actively form MCS between the pathogen-containing vacuole and the host ER, while MCS have not thus far been observed for *A. phagocytophilum* or *E. chaffeensis.*

MCS between a pathogen-containing vacuole and the ER is best understood for *C. trachomatis*. The *C. trachomatis* inclusion protein IncD directly binds to the plekstrin homology domain of CERT, a host ceramide transfer protein which binds VAP protein on the ER (Derre et al., 2011; Agaisse and Derre, 2015). IncD-CERT binding causes a conformational change to expose the CERT FFAT motif, enabling CERT to bind VAPA/B on the ER and form inclusion-ER MCS. Further, the sphingomyelin synthase proteins SMS1 and SMS2 are recruited to inclusion-ER MCS (Elwell et al., 2011). Given the importance of sphingomyelin in *C. trachomatis* development, it is hypothesized that IncD-CERT-VAP facilitate ceramide transport and conversion to sphingomyelin, which is then utilized by the bacteria (Elwell et al., 2011). *C. trachomatis* inclusion-ER MCS also contain the ER calcium sensor STIM, although STIM is not required for *C. trachomatis* growth and its role in MCS is not clear (Agaisse and Derre, 2015). A second Inc protein, CT005/IncV, also binds to VAP to mediate inclusion-ER MCS (Stanhope et al., 2017). IncV contains two FFAT motifs in the C-terminal tail; the first motif is similar to the canonical motif, while the second motif is non-canonical and contains a serine tract immediately upstream of IncV FFAT motif cores (Ende et al., 2022). Intriguingly, IncV-VAP interactions are regulated by phosphorylation of the serine tract of the noncanonical FFAT motif (Ende et al., 2022). The C-terminus recruits the host protein kinase CK2, which phosphorylates a serine tract upstream of the FFAT core, enabling binding to VAP. Thus, at least two *C. trachomatis* inclusion proteins facilitate interactions with the ER, highlighting the importance of this interaction.

During *C. burnetii* infection, electron tomography identified MCS of <5 nM between the CCV and host ER (Justis et al., 2017). Thus far, the host cell proteins ORP1L and VAPB have been identified in CCV-ER MCS, where VAPB is found on the ER and the ORP1L binds both the CCV and ER. ORP1L is a host sterol binding protein actively recruited to the CCV in a T4BSS-dependent manner during the first 24 hours of infection, although the ORP1L binding partner on the CCV is unknown (Justis et al., 2017). ORP1L is a member of the ORP family of sterol transfer proteins, many of which participate in MCS throughout the cell. The ORP1L ankyrin repeats are necessary and sufficient to localize ORP1L to the CCV, presumably through protein-protein interactions (Justis et al., 2017). ORP1L binds to ER-associated VAP proteins through the FFAT (two phenylalanines in an acidic tract) motif, thus facilitating MCS between the CCV and the ER. Like other members of the ORP family, ORP1L contains a lipid binding domain (OSBP-related domain, or ORD) which binds and transports a variety of lipids, including cholesterol, oxysterols, and phospholipids (Vihervaara et al., 2011). However, it is unknown whether ORP1L functions to transfer cholesterol between the CCV and ER, or rather facilitates MCS formation while other protein(s) are involved in lipid or small molecule transfer.

## Vacuole interactions with the cytoskeleton

The eukaryotic cytoskeleton, consisting of actin filaments, intermediate filaments and microtubules, is essential for maintaining the cell structure and internal organization, as well as cellular functions such as adhesion, vesicle transport, membrane traffic, division, and motility. Therefore, intracellular bacteria use a myriad of strategies to manipulate the host cytoskeletal machinery in order to successfully establish and sustain an intracellular infection. Of note, the bacteria internalization in non-phagocytic and phagocytic cells depends on cytoskeletal rearrangement at the site of bacterial entry.

Following host cell entry, internalized bacteria continue interacting with the cytoskeleton throughout the intracellular cycle. For instance, in *C. burnetii*-infected cells actin filaments are not only recruited but also involved in the formation of the CCV (Aguilera et al., 2009). Given that *C. burnetii* activates the host cyclic AMP-dependent protein kinase (PKA) during infection, it has been suggested that PKA regulates CCV biogenesis/expansion by modulating cytoskeleton-related proteins, including actin polymerization around the maturing vacuole (Macdonald et al., 2012; Macdonald et al., 2014). Indeed, the actin regulatory protein vasodilator-stimulated phosphoprotein (VASP) was identified as a PKA substrate that is increasingly phosphorylated during *C. burnetii* infection in a T4SS-dependent manner (Colonne et al., 2016). Importantly, optimal CCV formation, heterotypic fusion with other compartments, and bacterial replication depends on VASP activity, presumably because VASP transports vesicles along the cytoskeleton to the CCV (Colonne et al., 2016). Accordingly, filamentous actin patches on the CCV membrane requires the secretion of *C. burnetii* T4BSS effector proteins and serve as a scaffold for fusion of late endocytic vesicles and the CCV (Miller et al., 2018). Surprisingly, the CCV actin patches are not necessarily required for CCV biogenesis and stability, but the Arp2/3-mediated production of actin filaments that regulate trafficking within the endosomal system is essential for CCV formation and bacterial growth (Miller et al., 2018).

The intermediate filament protein vimentin, which is implicated in intracellular trafficking events (Styers et al., 2005), binds to the *C. burnetii* effector protein AnkF and is recruited to the CCV in a time-dependent manner (Pechstein et al., 2020). While vimentin is not required for bacterial replication, it appears to provide a platform for fusion and fission events, which also contributes to CCV formation (Pechstein et al., 2020). Similarly, the *A. phagocytophilum* toxin A (AptA) interacts with vimentin on the ApV membrane. Vimentin is required for activation of mammalian Erk1/2 mitogen activated protein kinase, which facilitates *A. phagocytophilum* survival within human neutrophils (Sukumaran et al., 2011). In addition to vimentin, the ApV is surrounded by the intermediate filament keratin (Truchan et al., 2016a). Whereas SUMO-2/3 colocalizes with both vimentin and keratin filaments, SUMOylation (a reversible post-translation modification where small ubiquitin-like modifier [SUMO] proteins are covalently attached to proteins by SUMO-specific enzymes) is only critical for the vimentin assembly at the ApV, and is important for optimal ApV formation and bacterial growth (Truchan et al., 2016a). SUMO2/3 proteins also surround the *E. chaffeensis*-containing vacuole (EcV), where they colocalize with the *E. chaffeensis* effector protein TRP120. TRP120 SUMOylation increases interaction with cytoskeletal host proteins, including γ-actin and myosin-X (also known as Myo10), which are involved in filopodium formation and microtubule cargo trafficking, respectively. Therefore, the enhanced interaction with these proteins may modulate actin rearrangement and affect cytoskeletal reorganization during *E. chaffeensis* infection (Dunphy et al., 2014). The *E. chaffeensis* effector protein TRP75 interacts with actin-binding or actin-related proteins, including ARPC5, LCP1, PLEK, and TPM4 (Luo et al., 2018), while the *E. chaffeensis* TRP47 interacts with the actin-binding protein CAP1 (Wakeel et al., 2009). While the mechanisms are unknown, associations between bacterial proteins with actin, actin-binding proteins and actin-related proteins during *E. chaffeensis* infection suggest that actin cytoskeleton reorganization might contribute to structural support and stabilization of EcVs during the entire intracellular bacterial life cycle.

Besides providing a structural support for cells, microtubules serve as a rail for vesicle trafficking through the cell, and the microtubule-based motor proteins, kinesins and dyneins, convey their intracellular cargos (Ross et al., 2008). Disruption of microtubules dynamics negatively affects CCV size and bacterial replication, as CCV biogenesis relies on the recruitment of the molecular machinery required for microtubule-dependent retrograde transport and tethering processes (Ortiz Flores et al., 2019). Interestingly, initial studies showed that microtubule networks are rapidly regenerated around the *C. trachomatis* inclusion after incubation with the microtubule-disrupting agent nocodazole (Campbell et al., 1989). Furthermore, the nascent *C. trachomatis* inclusion moves towards the minus end-directed microtubule motor dynein from the cell periphery to the microtubule-organizing center (MTOC), where it resides throughout *C. trachomatis* life cycle (Clausen et al., 1997; Grieshaber et al., 2003). This event is actively induced by the bacteria, as the *C. trachomatis* Inc CT850 interacts with the dynein light chain DYNLT1 to promote appropriate positioning of the inclusion at the MTOC (Mital et al., 2015). In addition, the *C. trachomatis* inclusion protein IPAM (inclusion protein acting on microtubules) interacts with the centrosomal protein CEP170 to orchestrate host microtubule reorganization at the inclusion periphery, allowing maintenance of inclusion shape to support bacterial intracellular development (Minikel et al., 1983). Importantly, microtubules encasing the inclusion can undergo different post-translational modifications (PTMs), which can influence their structure and depolymerization rates (Peris et al., 2009; Al-Zeer et al., 2014; Wesolowski et al., 2017). For instance, the C*. trachomatis* effector protein CT813/InaC recruits the host GTPases ARF1 and ARF4 to the inclusion membrane, where they induce post-translational modification of microtubules and Golgi complex positioning around the inclusion (Wesolowski et al., 2017). Thus, given the importance of the microtubule-based transport of the inclusion to *C. trachomatis* growth [reviewed in detail by (Nogueira et al., 2018)], other unidentified bacterial effector proteins are likely involved in this event. Finally, even though the mechanisms involving the association among the other cytoskeleton components with the *C. trachomatis* inclusion have not been fully elucidated yet, it is known that actin, as well as intermediate filaments, associate with the inclusion (Dong et al., 2004; Kumar and Valdivia, 2008; Chin et al., 2012). Formation of F actin at the inclusion depends on RhoA (Ras homolog family member A, a small GTPase), and its disruption leads to intermediate filaments disassemble, loss of inclusion integrity and leakage of inclusion contents into the host cytoplasm (Kumar and Valdivia, 2008).

The secreted bacterial protein CPAF (*C. trachomatis* protease/proteasome-like activity factor) is required for the cleavage of different intermediate filaments, including vimentin, keratin 8, keratin-18 (Dong et al., 2004; Kumar and Valdivia, 2008). It has been suggested that this event likely increases the solubility of these cytoskeletal structures to facilitate inclusion expansion (Dong et al., 2004; Savijoki et al., 2008). However, CPAF-cleaved vimentin, keratin-8 and keratin-18 remain morphologically as filamentous forms and retain their polymer functions (Kumar and Valdivia, 2008). Therefore, it is proposed that the intermediate filaments are progressively nicked by CPAF to form a highly dynamic actin/filament cage, which provides structure to accommodate exponential bacterial replication and inclusion expansion (Kumar and Valdivia, 2008).

## Egress/escape from the vacuole/host cell

**Figure 2:**
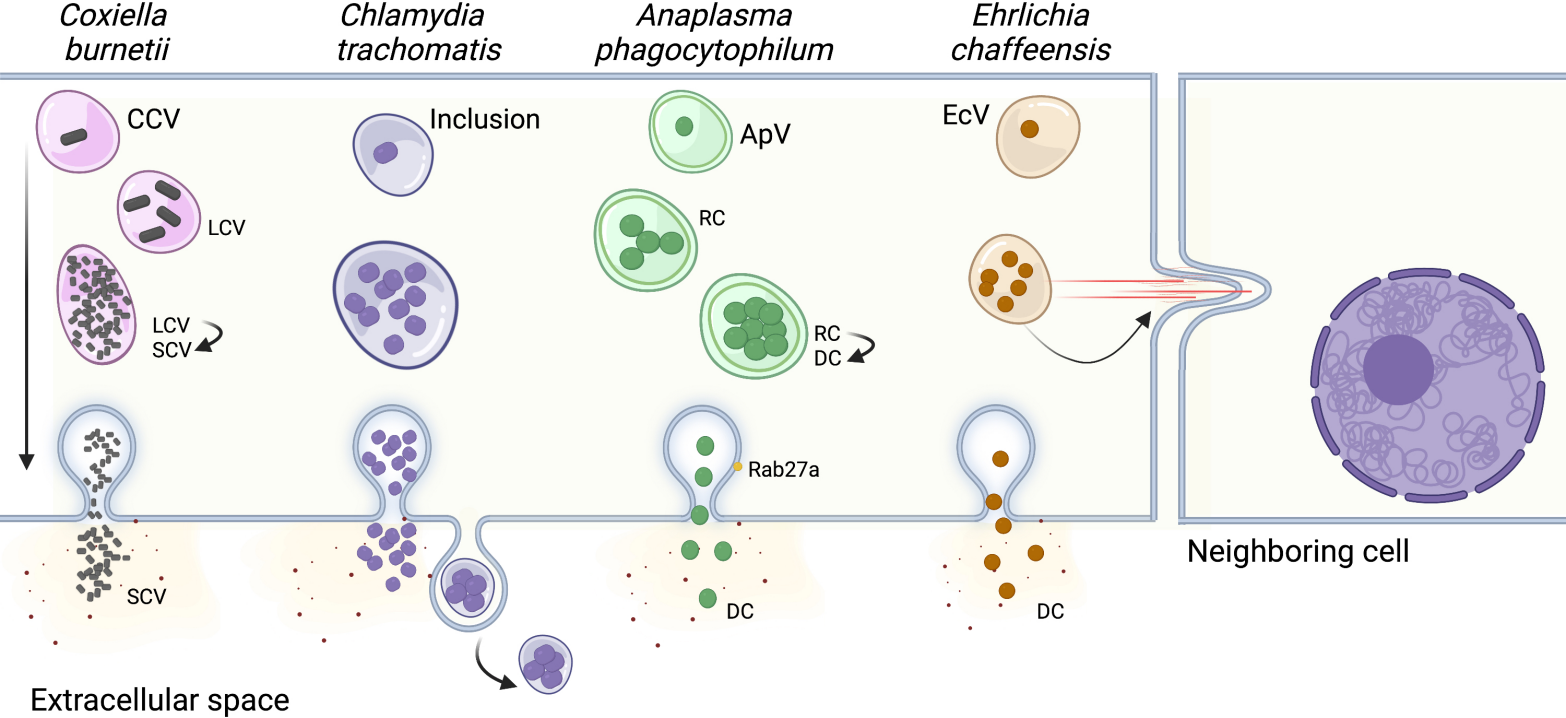
Pathogen escape from the host cell. In order to perpetuate an infection, most bacterial pathogens actively escape from the host cell to infect neighboring cells. The vacuoles harboring *Chlamydia trachomatis*, *Anaplasma phagocytophilum*, and *Ehrlichia chaffeensis* fuse with the host cell plasma membrane, releasing bacteria into the extracellular space. The *C. trachomatis* inclusion can also be directly released from the cell, similar to exosomes. *E. chaffeensis* utilizes a third mechanism of cell-to-cell spread, where the vacuole is transported directly to neighboring cells by filopodia. Finally, the mechanism of egress for *Coxiella burnetii* has not been identified and is thought to occur by spontaneous lysis of the host cell after the bacteria’s developmental cycle has been completed. CCV, *Coxiella* containing vacuole; LCV, *Coxiella* Large Cell Variant; SCV, *Coxiella* Small Cell Variant; ApV, *Anaplasma phagocytophilum* containing vacuole; RC, reticulate cell; DC, dense cell EcV, *Ehrlichia chaffeensis* containing vacuole. Created with Biorender.

During their intracellular life cycle, vacuolar pathogens are temporarily protected from the host immune defenses. However, to disseminate within the host, they must exit their host cells and successfully invade other cells to reinitiate the infection cycle. Exit from host cells can occur through a passive process, where the cells lyse due to a physical stress caused by a large number of replicating-pathogens, or it can be a complex process called “egress”, which relies on a dynamic interplay between host and pathogen factors (**Figure 2**).

Although it is unclear whether *C. burnetii* has developed specific strategies for host cell exit, *C. burnetii-*infected cells along with CCVs are spontaneously lysed after the replicative large cell variants (LCV) convert back to the infectious small cell variants (SCV), and the “naturally released” bacteria infect neighboring cells to start a new cycle of infection (Howe and Mallavia, 2000; Zhang et al., 2021). Similarly, *A. phagocytophilum* release to the extracellular environment precedes reinfection of nearby cells. This process involves the host exosome release pathway and is facilitated by the multivesicular body (MVB) proteins ALIX, ESCRT-III, Rab27a, and Munc13-4. The association of Rab27a with ApVs positioned at the plasma membrane promotes the release of bacteria into the extracellular environment. (Blouin and Kocan, 1998; Read et al., 2022). The bacterial protein APH1235 expression is pronouncedly upregulated at later time points of infection, correlating with transition from the noninfectious reticulate cell (RC) to the infectious dense-cored cell (DC) and subsequent DC exit from host cells. However, the specific role of this protein during bacterial egress has not yet been elucidated (Troese et al., 2011). *E. chaffeensis* can be transported to neighboring cells through filipodia formation during initial stages of infection, which allows cell-to-cell bacterial spread without exposing the bacteria to the host’s immune system in the extracellular space (Thomas et al., 2010). Interestingly, at later stages of infection, *E. chaffeensis* is also released by host cell membrane rupture adjacent to the EcV. However, the determinants dictating either exit route remain unknown (Thomas et al., 2010).

Like *E. chaffeensis, C. trachomatis* can escape the host cells by two different mechanisms, extrusion of the inclusion or host cell lysis (Beatty, 2007; Hybiske and Stephens, 2007). Early observations using scanning and transmission electron microscopy suggested that the *C. trachomatis* inclusion was transported to the host cell surface by a process similar to exocytosis, with the cells remaining intact but lacking a significant portion of their plasma membrane (Todd and Caldwell, 1985). Non-lytic *C. trachomatis* egress was later proved using live cell imaging, where approximately 50% of inclusions are indeed pinched off from the host cell by extrusion, and both inclusions and host cells remain intact (Hybiske and Stephens, 2007). This package release mechanism is independent of the microtubule network and conventional vesicular trafficking, but requires actin polymerization, N-WASP activity, myosin II, Rho GTPase, and septins (Hybiske and Stephens, 2007; Volceanov et al., 2014). Septins 2, 9, 11 and possibly 7, combined with F actin, form fibers that encase the inclusion. Depletion of individual septins by RNAi prevents F actin recruitment and fiber formation, reducing the number of extrusions. These findings indicate that septins are required for *C. trachomatis* release by extrusion and strengthen the role of actin in this process (Volceanov et al., 2014). Accordingly, actin is actively recruited by *C. trachomatis* effector protein(s) to the inclusion at 20 hours post-infection and increases in prevalence and extent throughout the *C. trachomatis* developmental cycle, culminating with their exit from the host cell by extrusion (Chin et al., 2012).

The remaining inclusions are released through host cell lysis, which involves rupture of both the inclusion and plasma membrane (Beatty, 2007; Hybiske and Stephens, 2007). During this event, disruption of the host cell plasma membrane and an influx of exogenous calcium precedes inclusion rupture (Hybiske and Stephens, 2007). In order to prevent complete host cell lysis, the plasma membrane is repaired by lysosomal exocytosis, which is regulated by elevated cytosolic calcium levels and actin depolymerization. Therefore, this lysosome-mediated repair process results in retention of residual bacteria within the surviving host cell, and release of several EBs capable of infecting other cells (Beatty, 2007). To exit their host cells using the lytic process, *C. trachomatis* must dismantle themselves from the cytoskeletal structures that encase its inclusion. Therefore, while the actin polymerization inhibitor latrunculin B blocks extrusion formation, it facilitates *C. trachomatis* lytic exit (Hybiske and Stephens, 2007; Yang et al., 2015). *C. trachomatis* lytic exit relies on bacterial proteins encoded on the *C. trachomatis* plasmid, as plasmidless *C. trachomatis* are incapable of disassemble actin from the inclusion, fail to exit cells and remain intracellular as mature inclusions yielding large numbers of infectious organisms (Yang et al., 2015).

The *C. trachomatis* Inc CT228 has been shown to play an important role during bacterial egress (Lutter et al., 2013; Shaw et al., 2018). It was previously suggested that CT228 preferentially recruits the phosphorylated form of MYPT1 (a subunit of myosin phosphatase) to the inclusion in order to inhibit its activity on MLC2 (myosin light chain 2) and facilitate extrusion-mediated exit (Lutter et al., 2013). However, new findings showed that loss of MYPT1 recruitment to the inclusion membrane, caused by CT228 disruption, significantly increases inclusion extrusion, suggesting that CTT28 inhibits extrusion. (Shaw et al., 2018). In addition, given the dramatic reduction in phosphorylation of MYPT1 at later stages of infection (Lutter et al., 2013), the MYPT1 recruitment to the inclusion can culminate in its activation overtime. Interestingly, CT228-mediated MYPT1 recruitment affects the longevity of infection *in vivo*, which may be related to the degree of host cell exit *via* extrusion (Shaw et al., 2018). As previously mentioned, extrusion formation is also regulated by interactions between the inclusion protein MrcA (or CT101) with host ITRP3 (calcium channel) and STIM1 (calcium sensor), as inhibition of extrusion is observed following siRNA depletion of ITPR3 or STIM1 or loss of ITPR3 recruitment due to MrcA disruption. Furthermore, inhibiting extrusion correlates with reduced phosphorylated MLC2, which is essential for myosin motor activity, and the intracellular calcium chelation by BAPTA-AM also reduces *C. trachomatis* extrusion (Nguyen et al., 2018). These findings reinforce the importance of calcium signaling pathways in regulation of release mechanisms by *C. trachomatis*.

## Concluding remarks

For obligate intracellular bacteria, survival requires an intracellular niche which both protects from the innate immune system and provides nutrients and other resources required for bacterial replication. Of the four vacuolar pathogens considered here, all have unique intracellular niches tailored to meet the specific requirements of each bacterium (summarized in **Table 1**). While significant progress has been made in our understanding of pathogen-containing vacuoles, many areas remain unanswered. This includes elucidation of both the protein and lipid profile of the pathogen containing vacuole membrane, how it changes during the course of infection, and the molecular mechanisms which regulate fusion with host vesicular trafficking. While membrane contact sites are emerging as critical players in nutrient exchange between the *Chlamydia* and *Coxiella* containing vacuoles and the host endoplasmic reticulum, their full composition and regulation are unknown, and it is not clear if membrane contact sites exist for other vacuole pathogens. Finally, while the host cytoskeleton is involved in all stages of infection, there is still a lack of understanding on how bacterial pathogens manipulate the cytoskeleton, particularly during pathogen escape. Recent advances in the genetics of obligate intracellular bacteria, as well as new techniques to analyze complex interactions at the molecular and cellular level, will facilitate a better understanding of how pathogens survive inside the host cell.

**Table 1:** Comparative analysis of key characteristics in the life cycles of *A. phagocytophilum*, *E. chaffeensis*, *C. burnetii*, and *C. trachomatis*.

	*A. phagocytophilum*	*E. chaffeensis*	*C. burnetii*	*C. trachomatis*
**Primary host cell**	Neutrophils	Monocytes and macrophages	Alveolar macrophages	Epithelial cells
**Transmission mode**	Ticks, blood transfusion	Ticks	Aerosols, contaminated dairy products	Sexual contact, mother to child
**Entry mechanism**	Caveolae and GPI-anchored proteins [P-selectin glycoprotein ligand 1 (PSGL-1)]	Caveolae and GPI-anchored proteins [GPI-anchored DNAse X]	αvβ3 integrin, other unknown host cell receptors	heparan sulfate proteoglycans (HSPGs), and multiple host cell receptors such as fibroblast growth factor receptor (FGFR), ephrin A2 receptor (EPHA2), and epithelial growth factor receptor (EGFR)
**Vacuole interactions with host vesicular trafficking**	autophagosome, recycling endosome, multivesicular body	Early endosome, early autophagosome	Late endosome, lysosome, autophagosomes	Golgi vesicles
**Homotypic fusion**	No	No	Yes	Yes
**Type of secretion system**	T1SS and T4SS	T1SS and T4SS	T4SS and sec-mediated secretion system	T3SS
**Vacuole acidity**	Moderately acidic	Moderately acidic	Moderately acidic	Neutral
**Exit mechanism**	MVB-exosome secretion pathway	host filopodia	Cell lysis	Active extrusion pathway, cell lysis

## Author contributions

All authors contributed to manuscript writing and revisions, and approved the submitted version
